# Biological Effects of XyloCore, a Glucose Sparing PD Solution, on Mesothelial Cells: Focus on Mesothelial-Mesenchymal Transition, Inflammation and Angiogenesis

**DOI:** 10.3390/nu13072282

**Published:** 2021-06-30

**Authors:** Valentina Masola, Mario Bonomini, Maurizio Onisto, Pietro Manuel Ferraro, Arduino Arduini, Giovanni Gambaro

**Affiliations:** 1 Division of Nephrology and Dialysis, Department of Medicine, Piazzale A. Stefani 1, 37126 Verona, Italy; giovanni.gambaro@univr.it; 2 Department of Biomedical Sciences, University of Padova, Viale G. Colombo 3, 35121 Padova, Italy; maurizio.onisto@unipd.it; 3 Nephrology and Dialysis Unit, Department of Medicine, G. d’Annunzio University, Chieti-Pescara, SS.Annunziata Hospital, Via dei Vestini, 66013 Chieti, Italy; mario.bonomini@unich.it; 4 U.O.S. Terapia Conservativa della Malattia Renale Cronica, U.O.C. Nefrologia, Dipartimento di Scienze Mediche e Chirurgiche, Fondazione Policlinico Universitario A. Gemelli IRCCS, 00178 Rome, Italy; pietromanuel.ferraro@unicatt.it; 5 Dipartimento Universitario di Medicina e Chirurgia Traslazionale, Università Cattolica del Sacro Cuore, 00178 Rome, Italy; 6 R&D Department, Iperboreal Pharma Srl, 65122 Pescara, Italy; a.arduini@iperboreal.com

**Keywords:** peritoneal dialysis, fibrosis, biocompatible solutions, L-carnitine, Xylitol

## Abstract

Glucose-based solutions remain the most used osmotic agents in peritoneal dialysis (PD), but unavoidably they contribute to the loss of peritoneal filtration capacity. Here, we evaluated at a molecular level the effects of XyloCore, a new PD solution with a low glucose content, in mesothelial and endothelial cells. Cell viability, integrity of mesothelial and endothelial cell membrane, activation of mesothelial and endothelial to mesenchymal transition programs, inflammation, and angiogenesis were evaluated by several techniques. Results showed that XyloCore preserves mesothelial and endothelial cell viability and membrane integrity. Moreover XyloCore, unlike glucose-based solutions, does not exert pro-fibrotic, -inflammatory, and -angiogenic effects. Overall, the in vitro evidence suggests that XyloCore could represent a potential biocompatible solution promising better outcomes in clinical practice.

## 1. Introduction

Peritoneal dialysis (PD) is one of the therapeutic options available for end-stage renal disease (ESRD) patients and represents an important alternative to hemodialysis (HD) [1]. PD offers more flexibility, allowing patients to continue working; it preserves their residual renal function and has a lower cardiovascular impact than HD [2,3,4].

On the other hand, continuous contact with glucose dialysis solutions during PD can induce significant morphological and functional changes in the peritoneum [5]. These include progressive sub-mesothelial thickening, narrowing and distortion of the vascular lumen with hyalinization, thickening of the basal capillary membrane, as well as thickening of the arterial wall, greater synthesis of pro-inflammatory cytokines and reactive oxygen species, inhibition of cell growth and proliferation, and DNA damage [6,7,8,9,10,11].

Mesothelial cells are arranged in a single layer in the peritoneum and they are able to regulate peritoneal inflammation and remodeling of the peritoneal tissue by secreting inflammation mediators, chemokines, growth factors and components of the ECM [12,13,14,15]. Furthermore, morphologically, mesothelial cells have characteristics in common with epithelial cells. Therefore, long-term exposure to PD fluids can cause morphological and functional changes similar to those that occur during epithelial-mesenchymal transition (EMT) [16,17,18], and consequently induce mesothelial to mesenchymal transition (MMT) [19].

In light of the above, the relationship between the mesothelium and dialysis solutions is fundamental in order to stem the hyper-activation of a pro-inflammatory, oxidative, and pro-fibrotic components.

In addition, it has recently been demonstrated that high concentrations of glucose are capable of inducing a significant increase in gene and protein expression of MMT markers [20]. Hence, the use of more biocompatible solutions could have an important positive impact on the regulation of the deranged pathways during PD treatment. Moreover, it has been shown that vascular endothelial growth factor (VEGF) is decidedly implicated in PD-associated angiogenesis, resulting in ultrafiltration failure [21,22]. In vivo studies have proven a link between increased VEGF production and acidic glucose-based PD solution [23]. Furthermore, AGEs are known to upregulate VEGF [15].

Many factors have been claimed as contributors to the poor biocompatibility of PD solutions, including high glucose content, elevated levels of glucose degradation products (GDPs) generated during heat-sterilization of glucose-based solutions, lactate buffer, acidic pH, and hyperosmolarity [24]. To overcome the un-physiology of peritoneal dialysis solutions, different approaches have been realized. So called biocompatible PD solutions, which are glucose-based but are characterized by neutral or physiological-pH and low GDP content using multi-chamber bags, were introduced into the market [23]. In these solutions, glucose is stored at very low pH to prevent GDP formation during heating and storage, and is separated from buffer (lactate and/or bicarbonate) and electrolytes. Mixture of the compartments before use results in a PDS with a more physiological pH. Despite several in vitro and experimental in vivo studies [25], however, recent findings suggest that improved biocompatibility of neutral-pH, low-GDP fluids cannot be assumed [26]. Indeed, in PD children treated with such dialysates for four months, peritoneal membrane biopsies showed early peritoneal inflammation, fibroblast activation, increased VEGF and vessel density, which affected the PD membrane transport function [27].

A different approach is represented by the replacement of glucose in PDS with alternative osmotic agents. Several alternatives to glucose were evaluated over the years but only two osmotic agents are currently available in glucose-free dialysate for PD clinical practice: icodextrin and amino acids. Icodextrin is a water-soluble glucose polymer derived from starch, which is indicated for use during a single long dwell per day being associated with a slow but sustained peritoneal ultrafiltration [28]. Amino acid-containing PD fluid gives the possibility of improving the nutritional status of some malnourished PD patients [29]. Potential benefits of glucose-sparing by above formulations have been reported [30]. The use of such dialysates, however, is limited to as a single daily peritoneal exchange and replace no more than 30–50% of the glucose absorbed every day [31]. Moreover, direct knowledge of their impact on the peritoneal membrane is lacking, since no biopsies of peritoneal tissues have been obtained so far from PD patients on icodextrin or amino acid solutions [25].

In this scenario, new PD solutions in the developing phase are very promising, in particular those containing Xylitol and L-Carnitine [31,32].

L-Carnitine is a naturally occurring compound involved in mitochondrial oxidation of long-chain fatty acids [33]. It is water soluble and has osmotic properties. Animal studies have shown that equi-osmolar solutions of L-Carnitine and glucose are equally active on peritoneal ultrafiltration [33], but with a better biocompatible profile as shown in in-vitro and in vivo studies [34,35]. A recent clinical study, while confirming the safety of L-Carnitine, has also shown an improvement in insulin sensitivity among non-diabetic ESRD patients on PD therapy [36].

Xylitol is a five-carbon sugar alcohol derived by the reduction of D-xylulose. Once inside the cell, D-Xylitol is oxidized to D-xylulose and then phosphorylated to D-xylulose-5-phosphate, an intermediate of the pentose monophosphate shunt [31]. Xylitol can act as an osmotic agent [37], improves insulin sensitivity and glycemic control in diabetic patients [31,37], and has a good biocompatibility and toxicity profile [32,38]. In Germany, xylitol is approved for parenteral nutrition.

Interestingly, at odds with glucose that generates GDPs and AGE when steam-sterilized, both L-carnitine and xylitol are extremely stable and heat resistant at temperatures used for steam-sterilization of PD solutions [39,40].

In view of these encouraging premises, two clinical trials (NCT04001036 and NCT03994471) testing the association of L-carnitine, Xylitol and low glucose are under way. The aim of the present study was to further investigate the effects of these new solutions at cellular level on mesothelial and endothelial cells.

## 2. Materials and Methods

### 2.1. Cell Culture

Human peritoneal mesothelial cells (HMRSV5), kindly obtained by Prof. Pierre Ronco (Paris), were cultured in DMEM/F12 (16.92 mM glucose) supplemented with 10% (*v*/*v*) heat-inactivated fetal bovine serum and 100 U/mL penicillin/streptomycin (Invitrogen) and growth in type I collagen coated plastics.

MeT5α cells (ATCC^®^ CRL-9444™), from an immortalized human mesothelial cell line, were maintained in M199 (5.37 mM glucose) with 10% fetal bovine serum (FBS), EGF (3.3 nM), insulin (860 nM), trace elements B, hydrocortisone (400 nM), and an antibiotic solution.

HUV-EC-C (HUVEC) (ATCC^®^ CRL-1730™) a human umbilical vein/vascular endothelium cell line, was cultured in F12K (7 mM glucose) medium, 10% FBS, 0.1 mg/mL heparin, and endothelial cell growth supplement.

HMVEC, a human microvascular endothelial cell line, was purchased from Lonza and maintained in endothelial growth medium (EGM™-2MV BulletKit™; Clonetics) (5.37 mM) supplemented with 5% FBS.

Cells were maintained in a humidified environment containing 5% CO_2_ at 37 °C, and the culture medium was replaced every 2 days. Cells were permitted to attach for 24 h and to grow to 80% confluence. Cells were also seeded and cultured on a polyester filter (0.4-lm pore size; Transwell, 12 well type, Millipore), using complete medium. The inner and outer chambers were filled with 0.5 and 1.5 mL of the culture medium, respectively, and the culture medium was replaced every 2 days.

### 2.2. Treatments

The different cell types were treated with Low Strength (LS) and Medium Strength (MS) XyloCore PD solutions (Iperboreal Pharma, Pescara, Italy), and 1.36% and 2.27% glucose-based Physioneal 40 PD solutions (Baxter, Italy). The detailed composition of tested solutions is described in Table 1.

Generally speaking, previous in-vitro studies on PD solution biocompatibility were carried out on cells grown on plastic with the PD solution diluted at a certain rate with cell medium. This was a compromise to maintain cell nutrient but that actually does not represent what really happens during PD: mesothelial cells are exposed to pure PD solution on the apical side and remain attached on the basal side whereas endothelial cells come in contact with PD fluids from the basolateral side. We performed our experiments on a culture model that reproduces conditions similar to in vivo ones. Consequently, we evaluated Physioneal versus XyloCore on cells grown on plastic and exposed to pure, undiluted PD solutions (though for a short time, 3 h) or grown to transwells and exposed to pure PD solutions on the apical side and to culture medium on the basal side. Subsequently, cells were recovered in new cell medium (both in apical and basolateral side).

### 2.3. Viability Assay

Mesothelial (HMRSV5 and MET5α) and endothelial (HMVEC and HUVEC) cells viability was assessed after 3 h of exposure to different PD solutions. Cells were plated on 96-well plates and transwells in complete medium, washed once with PBS and then treated for 3 h with serum free medium (CTR), Physioneal (1.36% and 2.27% glucose), and XyloCore (LS and MS). Cell viability was ascertained after the various treatments using the CellTiter 96 Aqueous One Solution Cell Proliferation Assay (3-(4.5-dimethylthiazol-2-yl)-5-(3-carboxymethoxyphenyl)-2-(4-sulfophenyl)-2H-tetrazolium, inner salt; MTS) (Promega), according to the manufacturer’s protocol. Briefly, 20 μL of reagent were added to each well, the plate was incubated for 3 h at 37 °C, then the absorbance was measured at 490 nm. Results were normalized to CTR which was set equal to 1.

### 2.4. Transepithelial Resistance and Albumin Permeability

Cells were cultured in transwells and transepithelial resistance (TER) was measured daily using a Millicell-ERS ohmmeter with electrodes (Millipore) inserted on both sides of the filter. The alternating current applied between the electrodes was within 620lA at a frequency of 12.5 Hz. The resistance of the monolayer was multiplied by the effective surface area to obtain the electrical resistance of the monolayer (X cm^2^). The background TER of the blank Transwell filter was subtracted from the TER of the cell monolayer. Once stable resistances had been obtained, different solutions were tested for 3 h, and then cells were recovered in complete medium. TER was measure 21 h after.

The permeability of the mesothelial monolayer was recorded after stable resistances had been obtained. Cells were treated with PD solutions and then Evans blue-labelled albumin (stock solution prepared with 1% Evans Blue and 5% Bovine Serum Albumin in PBS) was added to the inner chamber of the Transwell at a final concentration of 0.5 mg/mL. Samples were collected from the lower chamber 21 h after. Color changes were measured spectrophotometrically at 610 nm and the results are expressed as the percentage change in albumin permeability with respect to control.

### 2.5. Gene Expression Analysis

For gene expression analysis mesothelial and endothelial cells were plated (2 × 10^5^ cells/cm^2^) in transwells and when a stable transepithelial resistances had been obtained the medium was removed, cells were washed with PBS and then treated for 3 h in PD or control solution and then re-filled with medium for 24 h. Total RNA was extracted using the Trizol reagent (Invitrogen, Waltham, MA, USA), according to the manufacturer’s instructions. Yield and purity were checked using Nanodrop (EuroClone, Pero, Italy), and total RNA from each sample was reverse-transcribed into cDNA using SuperScript II Reverse Transcriptase (Invitrogen). Real-time PCR was performed on an ABI-Prism 7500 using Power SYBR Green Master Mix 2X (Applied Biosystems, Waltham, MA, USA). The comparative Ct method (DDCt) was used to quantify gene expression, and the relative quantification was calculated as 2^−DDCt^. The presence of non-specific amplification products was excluded by melting curve analysis. The primers used are listed in Table 2.

### 2.6. Western Blotting

For protein expression analysis mesothelial and endothelial cells were plated (2 × 10^5^ cells/cm^2^) in transwells and when a stable transepithelial resistances had been obtained the medium was removed, cells were washed with PBS and then treated for 3 h in PD or control solution and then re-filled with medium for 24 h. Cells were lysed in 50 mM Tris–HCl, pH 5.0, 150 mM NaCl, 0.5% Triton X-100 with Complete Protease Inhibitor Mixture (Roche Applied Science, Penzberg, Germany). Briefly, equal amounts of proteins were treated in reducing sample buffer and denatured for 10 min at 100 °C. Protein samples were then resolved in 10% SDS–PAGE and electrotransferred to nitrocellulose membranes. Non-specific binding was blocked for 1 h at room temperature with non-fat milk (5%) in TBST buffer (50 mM Tris–HCl, pH 7.4, 150 mM NaCl and 0.1% Tween 20). Membranes were exposed to primary antibodies (1:1000) directed against ACTIN (sc-47778), VIMENTIN (VIM)(sc-7557), IL-1b(sc-23459) (Santa Cruz, Dallas, TX, USA), E-CADHERIN (E-CAD) (GTX10443 GeneTex, Irvine, CA, USA) and α-SMA (A5228 Sigma, St. Louis, MO, USA), overnight at 4 °C and incubated with a secondary peroxidase-conjugated antibody for 1 h at room temperature. The signal was detected with Luminata™ Forte Western HRP Substrate (Millipore) according to the manufacturer’s instructions and the signal was acquired with Mini HD9 (UVItec, Cambridge, UK).

### 2.7. Measurement of Mitochondrial ROS

MET5α cells were treated on plastic for 3 h in PD or control solution. After treatment, cells were incubated for 30 min in 5 μM MitoSOX (Life Technologies, Carlsbad, CA, USA) diluted with medium without phenol red. Cells were then rinsed with PBS and placed in fresh medium without phenol red. The fluorescence intensity of MitoSOX was measured by a microplate reader at excitation and emission wavelengths of 510 and 580 nm, respectively [41].

### 2.8. VEGF Secretion

HMRSV5 mesothelial and HUVEC endothelial cells were grown in transwells and when a stable transepithelial resistances had been obtained, the medium was removed, cells were washed with PBS, treated for 3 h in PD or control solution, then refilled with serum free medium for 24 h. Supernatants were collected, centrifuged, and stored at −20 °C until required. Analysis of VEGF in the supernatants was performed using standard ELISA method (Human VEGF-A ELISA Kit, SigmaAldrich) according to the manufacturer’s instructions. All experiments were performed in triplicate.

### 2.9. Angiogenesis Assay

Endothelial cell differentiation on Matrigel assay was performed following the method of Zimrin et al. [42]. A 48-well plate was filled with 100 μL/well of growth factor reduced matrigel (5 mg/mL) and allowed to polymerize for 1 h at 37 °C. HUVEC cells (3 × 10^4^ cells/well) were seeded into wells suspended in conditioned media of mesothelial cells (obtained as described in Section 2.7). Endothelial cells were then cultured for 5 h at 37 °C in a humidified atmosphere. Image analysis of the cell pattern was carried out using QWin image analysis software, as previously detailed [43], and the dimensional (area (%) and length (mm/field)) and topological (meshes and branching (number/field)) parameters were expressed as variations over the control (arbitrary units (AU) = 1). Images are representative of triplicates.

### 2.10. Statistical Analysis

Statistical analyses on real time RT-PCR data were performed using the Relative Expression Software Tool (REST). For comparison between two distributions, the two-tailed *t*-test was used. For multiple comparisons, one-way analysis of variance (ANOVA) was used with Sidak’s test (for multiple comparisons) using GraphPad Prism version 8.0 for Windows. *p* < 0.05 was considered significant for all tests.

## 3. Results

### 3.1. XyloCore Has a Lower Impact on Mesothelial and Endothelial Cell Viability than Do Commercial PD Solutions

**Figure 1:**
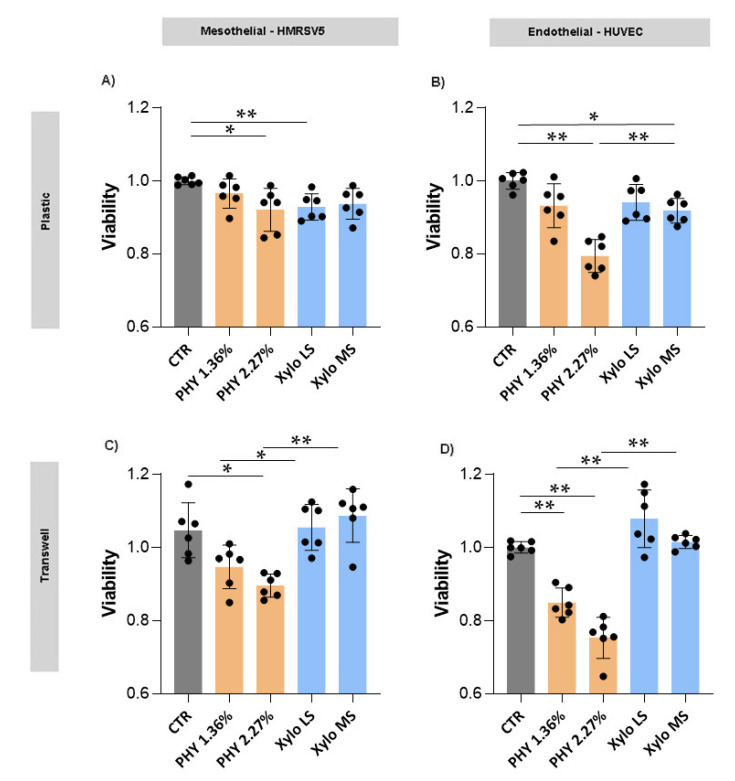
Cells viability. Cell viability was assessed with the MTS assay in mesothelial (**A**,**C**) and endothelial (**B**,**D**) cells cultured in plastic (**A**,**B**) or transwell (**C**,**D**) with or without different PD solutions. Graphs represent mean ± standard deviation (error bars) (*n* = 6 biological replicates). (**A**) CTR vs. PHY 2.27% *p* = 0.0148, CTR vs. Xylo LS *p* = 0.0337; (**B**) CTR vs. PHY 2.27% *p* < 0.0001, CTR vs. Xylo MS *p* = 0.0208, PHY 2.27% vs. Xylo MS *p* = 0.0003; (**C**) CTR vs. PHY 2.27% *p* = 0.0018, PHY 1.36% vs. Xylo LS *p* = 0.0363, PHY 2.27% vs. Xylo MS *p* = 0.0001; (**D**) CTR vs. PHY 1.36% *p* < 0.0001, CTR vs. PHY 2.27% *p* < 0.0001, PHY 1.36% vs. Xylo LS *p* < 0.0001, PHY 2.27% vs. Xylo MS *p* < 0.0001 * *p* < 0.05, ** *p* < 0.001.

HMRSV5 mesothelial and HUVEC mesothelial cells seeded on plastic showed a viability modulation when exposed for three hours to glucose-based PD solution. In detail, we observed a significative reduction of viability in cells treated with 2.27% glucose-based solution. The treatment of HMRSV5 and HUVEC cells with XyloCore (LS and MS) produced a mild viability reduction (Figure 1A,B). Similar effects were observed in MET5α mesothelial and HMVEC endothelial cells (Supplementary Figure S1A,B).

When HMRSV5 mesothelial and HUVEC endothelial cells, seeded on transwell, were treated for 3 h with glucose-based PD solutions, a reduction in viability of about 20% was found (Figure 1C,D). The mesothelial MET5α and endothelial HMVEC cells, treated on transwell supports, showed a similar behavior (Supplementary Figure S1C,D).

Overall results demonstrated that Physioneal significantly reduces mesothelial and endothelial cell viability, whereas Xylocore, independently of osmotic strength, does not induce significant modification in terms of cell viability after 3 h of treatment.

**Figure 2:**
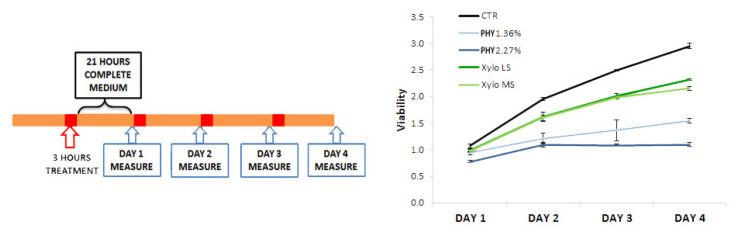
Cell viability over time. On the right: Schematic representation of the time-course viability assay. On the left: Graph represents mesothelial cell seeds on plastic viability assessed by MTS assay with or without different PD solutions at different time points. Dots represent mean ± standard deviation (error bars) (*n* = 6 biological replicates).

Since clinical PD is characterized by repeated cycles of peritoneum exposure to PD solutions, we evaluated mesothelial cell viability after consecutive treatments with PD solutions. HMRSV5 mesothelial cells were seeded on plastic in complete medium, treated for 3 h in PD or control solution, and then refilled with complete medium for 21 h (1st cycle). At this time, cell viability was assessed and cells were exposed up to a further 3 treatments (Figure 2). Results showed that the repetitive treatment cycles with glucose-based PD solutions significantly reduced mesothelial cell viability compared with controls and this was proportional to the number of exposures (Figure 2). However, it cannot be excluded that the viability reduction over time could be due to a reduced proliferation rate. On the contrary, repetitive use of XyloCore maintains the cell viability profile more similar to untreated cells (Figure 2).

### 3.2. Effect of PD Solution on Mesothelial and Endothelial Cell Morphology

Mesothelial to mesenchymal transition (MMT) of mesothelial cells and endothelial to mesenchymal transition of endothelial cells (EndoMT) are associated with morphological changes.

**Figure 3:**
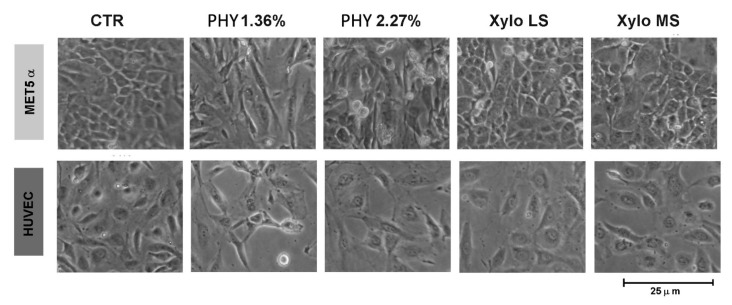
Morphological changes induced by PD solutions. Representative images of morphological changes at optical microscopy of mesothelial (Upper) and endothelial (Lower) cells treated for 3 h in PD or control solution and then recovered with complete medium for 24 h.

We observed that control cells exhibit a flat epithelial/endothelial aspect whereas a single treatment with glucose-based PD solutions modified cellular morphology. Specifically, cells presented elongated and loosed cell-cell contacts (Figure 3). Cells treated with XyloCore maintained a near normal morphology (Figure 3).

### 3.3. Effect of PD Solution on Mesothelial Trans-Epithelial Resistance (TER) and Mesothelial Permeability

TER and permeability to albumin were measured in our in-vitro model to see how high glucose and different PD solutions might also influence the mesothelial layer ultrafiltration function.

**Figure 4:**
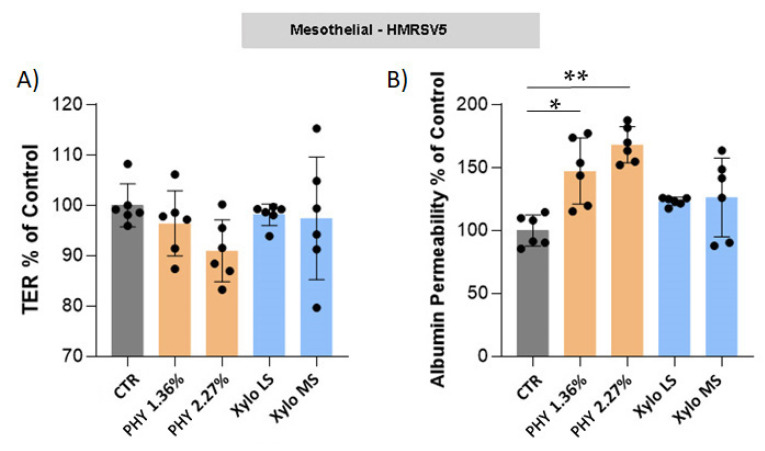
Mesothelial TER and permeability. (**A**) TER and (**B**) albumin permeability were measured in HMRSV5 mesothelial cells grown with or without different PD solutions. Graphs represent mean ± standard deviation (error bars) (*n* = 6 biological replicates). (**B**) CTR vs. PHY 1.36% *p* = 0.0025, CTR vs. PHY 2.27% *p* < 0.0001. * *p* < 0.05, ** *p* < 0.001.

Glucose-based PD solutions reduced the TER of the HMRSV5 mesothelial cell layer (Figure 4A) and significantly increased its albumin permeability (Figure 4B). A similar effect was also observed in MET5α mesothelial cells (Supplementary Figure S2A,B) (*n* = 4). XyloCore treatment preserved mesothelial layer integrity: TER and albumin permeability did not show differences compared with control cells (Figure 4 and Supplementary Figure S2A,B).

### 3.4. Effects of PD Solutions on Mesothelial and Endothelial Transdifferentiation

We investigated the effect of the exposure of mesothelial and endothelial cells to different PD solutions on their phenotype. First, we investigated the gene expression of transforming growth factor-β1 (TGF-β), the pivotal factor supervising fibrosis, and SNAIL, a TGF-β dependent transcription factor that activates EMT and EndoMT programs.

**Figure 5:**
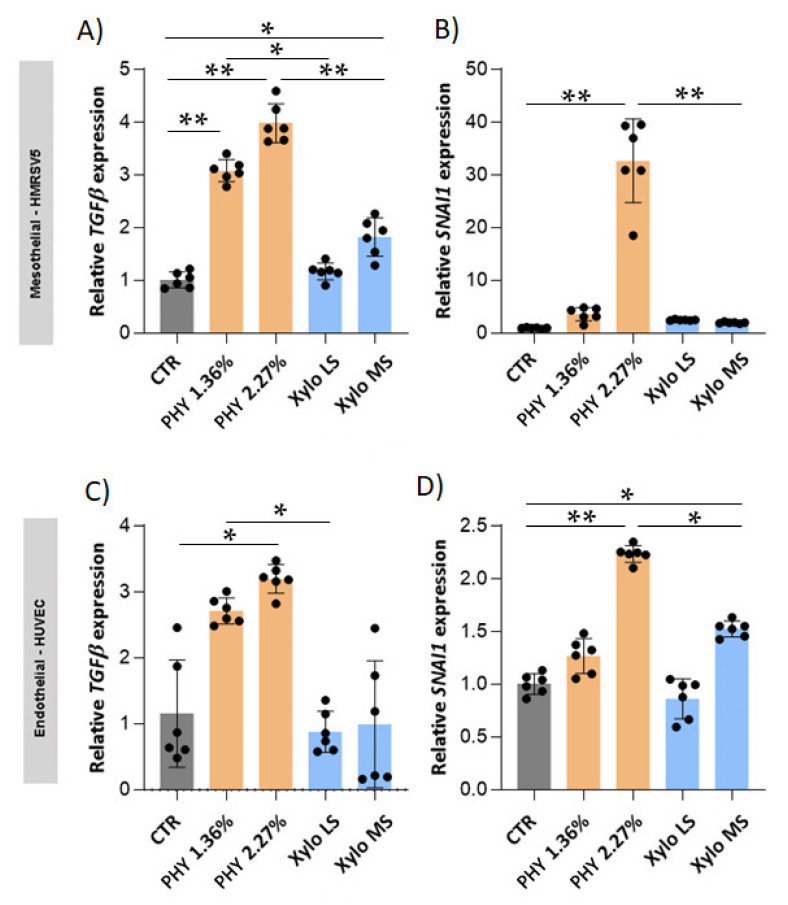
*SNAI1* and *TGF-β* expression in mesothelial and endothelial cells. *TGF-β* (**A**,**C**) and *SNAI1* (**B**,**D**) gene expression was quantified by real-time PCR. The analysis was performed in HMRSV5 mesothelial cells (**A**,**B**) and in HUVEC endothelial cells (**C**,**D**) treated for 3 h in PD or control solution and then recovered with complete medium for 24 h. The results were normalized using *ACTIN* as an internal control and represent the mean ± S.D. (error bars) (*n* = 6 biological replicates). (**A**) CTR vs. PHY 1.36% *p* < 0.0001, CTR vs. PHY 2.27% *p* < 0.0001, CTR vs. Xylo MS *p* = 0.0026, PHY 1.36% vs. Xylo LS *p* = 0.0019, PHY 2.27% vs. Xylo MS *p* < 0.0001; (**B**) CTR vs. PHY 2.27% *p* < 0.0001, PHY 2.27% vs. Xylo MS *p* < 0.0001; (**C**) CTR vs. PHY 2.27% *p* = 0.0053, PHY 1.36% vs. Xylo LS *p* = 0.0016; (**D**) CTR vs. PHY 2.27% *p* < 0.0001, CTR vs. Xylo MS *p* = 0.0095, PHY 2.27% vs. Xylo MS *p* = 0.0016. * *p* < 0.05, ** *p* < 0.001.

Results showed that glucose-based PD solutions significantly increase TGF-β expression and its downstream transcription factor *SNAI1* both in HMRSV5 mesothelial (Figure 5A,B) and in HUVEC endothelial cells (Figure 5C,D). Similar expression patterns were observed in MET5α mesothelial cells (Supplementary Figure S3A,B) and HMVEC endothelial cells (Supplementary Figure S3C,D). On the contrary, at equal osmotic strengths, *TGF-β* and *SNAI1* expressions, both in mesothelial and endothelial cells, were not influenced by XyloCore LS treatment (Figure 5 and Supplementary Figure S3). Similarly, XyloCore MS do not influenced *TGF-β* and *SNAI1* expression in MET5α mesothelial cells (Supplementary Figure S3A,B) and HMVEC endothelial cells (Supplementary Figure S3C,D). In HMRSV5, mesothelial (Supplementary Figure S5A,B) XyloCore MS mildly increased *TGF-β* but not *SNAI1* expressions. In HUVEC endothelial cells (Figure 5C,D), XyloCore MS mildly increased *SNAI1* but not *TGF-β* expression.

**Figure 6:**
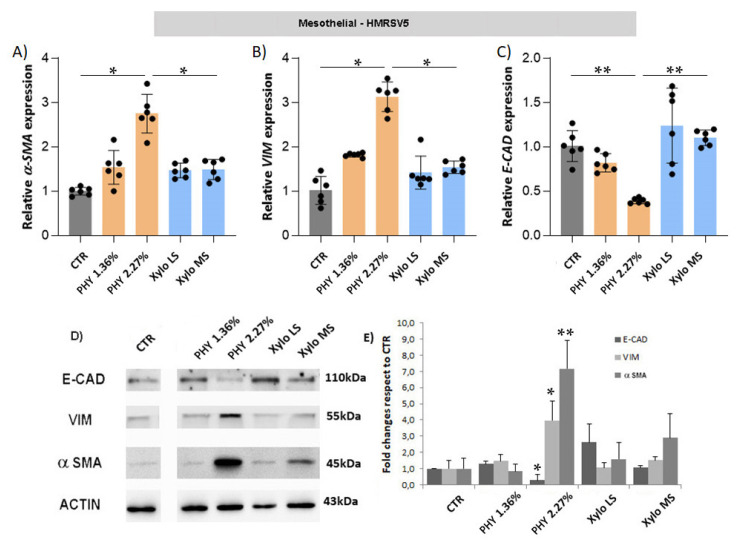
Expression of epithelial and mesenchymal markers in mesothelial cells. *α-SMA* (**A**), *VIM* (**B**) and *E-CAD* (**C**) gene expression. The analysis was performed in HMRSV5 mesothelial cells as described in Figure 5. (*n* = 6 biological replicates) (**A**) CTR vs. PHY 2.27% *p* = 0.0212, PHY 2.27% vs. Xylo MS *p* = 0.0173; (**B**) CTR vs. PHY 2.27% *p* = 0.0169, PHY 2.27% vs. Xylo MS *p* = 0.0033; (**C**) CTR vs. PHY 2.27% *p* = 0.0002, PHY 2.27% vs. Xylo MS *p* < 0.0001 (**D**) The protein expression of E-CAD, α-SMA and VIM was evaluated by Western Blot analysis. ACTIN was included as loading control. (**E**) WB quantification expressed as fold changes of bands intensity, normalized to ACTIN, respect to CTR (*n* = 3 biological replicates). Graph represent mean ± standard deviation (error bars). * *p* < 0.05, ** *p* < 0.001.

Activation of the MMT program is characterized by the expression of mesenchymal markers and the down-regulation of epithelial protein. HMRSV5 mesothelial cells exposed to glucose-based PD solutions exhibited a significant up-regulation of mesenchymal markers *α-SMA* and *VIM*, as well as *E-CAD*, a key epithelial marker, both at gene (Figure 6A–C) and protein (Figure 6D) levels. The treatment with XyloCore only slightly increased the expression of mesenchymal markers without affecting *E-CAD* expression. A comparable response was observed in MET5α mesothelial cells (Supplementary Figure S4).

**Figure 7:**
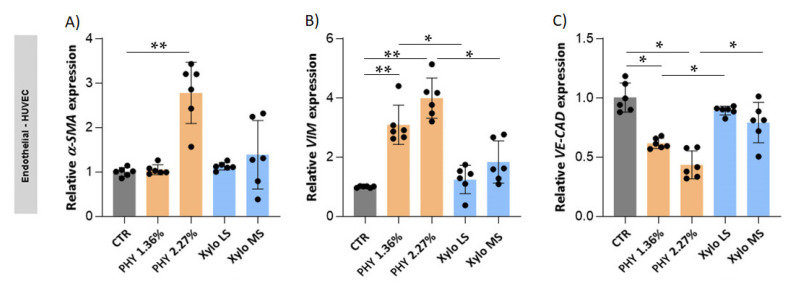
Expression of endothelial and mesenchymal markers in endothelial cells. *α-SMA* (**A**), *VIM* (**B**) and *VE-CAD* (**C**) gene expression. The analysis was performed in HUVEC endothelial cells as described in Figure 5, Graphs represent mean ± standard deviation (error bars)(*n* = 6 biological replicates) (**A**) CTR vs. PHY 2.27% *p* < 0.0001, (**B**) CTR vs. PHY 1.36% *p* < 0.0001, CTR vs. PHY 2.27% *p* < 0.0001, PHY 1.36% vs. Xylo LS *p* = 0.0086, PHY 2.27% vs. Xylo MS *p* = 0.0023, (**C**) CTR vs. PHY 1.36% *p* = 0.0019, CTR vs. PHY 2.27% *p* = 0.0016, PHY 1.36% vs. Xylo LS *p* = 0.0013, PHY 2.27% vs. Xylo MS *p* = 0.0036 * *p* < 0.05, ** *p* < 0.001.

Exposure of endothelial cells to high glucose concentration is able to activate EndoMT cells, but recent findings confirmed that this phenomenon also happens in sub-mesothelial vessels of long-term PD patients [44]. Glucose-based PD solutions, especially at 2.27% of glucose, unlike XyloCore, significantly upregulated the gene expression of mesenchymal markers *α-SMA* and *VIM* with concurrent down-regulation of the endothelial marker *VE-CAD* in HUVEC (Figure 7) and HMVEC endothelial cells (Supplementary Figure S5). On the contrary, XyloCore did not activate the EndoMT process.

### 3.5. Effects of PD Solutions on Peritoneal Inflammation and Mitochondrial Oxidative Stress

**Figure 8:**
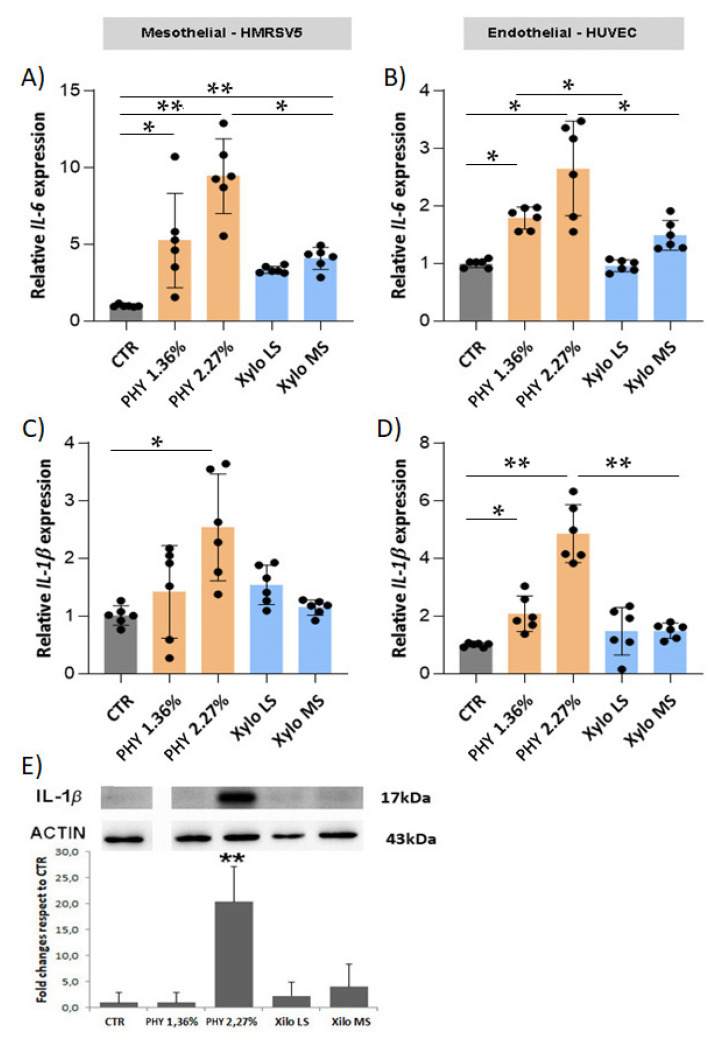
Expression of inflammatory markers in mesothelial and endothelial cells. *IL-6* (**A**,**B**) and *IL-1β* (**C**,**D**) gene expression. The analysis was performed as described in Figure 5. (**A**) CTR vs. PHY 1.36% *p* = 0.0021, CTR vs. PHY 2.27% *p* < 0.0001, CTR vs. Xylo MS *p* = 0.0009, PHY 2.27% vs. Xylo MS *p* = 0.0021; (**B**) CTR vs. PHY 1.36% *p* = 0.0122, CTR vs. PHY 2.27% *p* = 0.0036, PHY 1.36% vs. Xylo LS *p* = 0.0079, PHY 2.27% vs. Xylo MS *p* = 0.0022; (**C**) CTR vs. PHY 2.27% *p* = 0.0066; (**D**) CTR vs. PHY 1.36% *p* = 0.0482, CTR vs. PHY 2.27% *p* < 0.0001, PHY 2.27% vs. Xylo MS *p* < 0.0001 * *p* < 0.05, ** *p* < 0.001. (*n* = 6) (**E**) Upper: Protein expression of active-IL-1β was evaluated by Western Blot analysis. ACTIN was included as loading control. WB quantification expressed as fold changes of bands intensity, normalized to ACTIN, respect to CTR (*n* = 3 biological replicates). Graph represents mean ± standard deviation (error bars).

A micro-inflammatory state in PD patients potentially sustains the pro-angiogenic response [45] and the main cytokines involved are IL-1b, Il-6, and TNF-α [46]. Results showed that mesothelial and endothelial cells up-regulate the gene expression of pro-inflammatory cytokines *IL-6* and *IL-1β* when exposed to glucose-based PD solutions (Figure 8). Moreover, IL-1β protein was increased by the Physioneal 2.27% treatment in mesothelial cells. By contrast, treatment with XyloCore LS did not affect IL-6 and IL-1β gene and protein expression. XyloCore MS did not affect IL-1β gene and protein expression. It did not affect *IL-6* gene expression in endothelial cells and produced an increase of *IL-6* gene expression in mesothelial cells, which, however, was significantly lower in respect of Physioneal with comparable osmotic strength.

Oxidative stress has an important role in in the peritoneal failure during PD [47] and it has been demonstrated that high glucose PD solutions could be responsible of higher levels of oxidative stress [48]. Mitochondria represent the main source of oxidative stress and it has been recently demonstrated that mitochondrial ROS are involved in the EMT of mesothelial cells in the course of PD [49]. Results showed that Physioneal 2.27% significantly increased mitochondrial ROS, whereas XyloCore exerted no effects (Supplementary Figure S6).

### 3.6. Effects of PD Solutions on Peritoneal Angiogenesis

**Figure 9:**
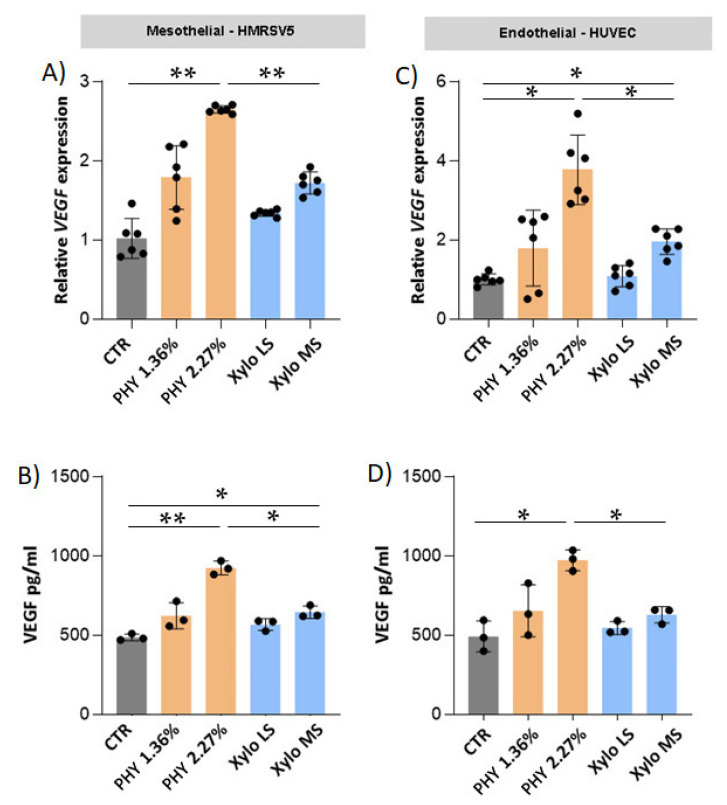
Expression and release of VEGF by endothelial and mesenchymal cells. (**A**,**C**) *VEGF* gene expression. The analysis was performed as described in Figure 5. (**A**) CTR vs. PHY 2.27% *p* < 0.0001, PHY 2.27% vs. Xylo MS *p* < 0.0001; (**C**) CTR vs. PHY 2.27% *p* = 0.0056, CTR vs. Xylo MS *p* = 0.0092, PHY 2.27% vs. Xylo MS *p* = 0.0078 * *p* < 0.05, ** *p* < 0.001. (*n* = 6 biological replicates) VEGF soluble protein was quantified in conditioned medium obtained after 24 h of incubation of HMRSV5 mesothelial cells (**B**) and in HUVEC endothelial cells (**D**) pre-treated for 3 h in PD or control solution. Values are given as mean ± SD. (**B**) CTR vs. PHY 2.27% *p* < 0.0001, CTR vs. Xylo MS *p* = 0.0158, PHY 2.27% vs. Xylo MS *p* = 0.0012; (**D**) CTR vs. PHY 2.27% *p* = 0.0066, PHY 2.27% vs. Xylo MS *p* = 0.0076. * *p* < 0.05, ** *p* < 0.001 (*n* = 3 biological replicates).

Physioneal significantly and dose-dependently increased *VEGF* gene expression and soluble released VEGF in both mesothelial (Figure 9A,B) and endothelial (Figure 9C,D) cells. On the contrary, XyloCore LS had no significant effects on VEGF expression and production in both mesothelial and endothelial cells. XyloCore MS did not influenced *VEGF* gene expression on mesothelial cells and VEGF release in endothelial cells, XyloCore MS increased *VEGF* gene expression on endothelial cells and VEGF release in mesothelial cells. However, the levels were significantly lower in respect of Physioneal with comparable osmotic strength.

Since the main source of VEGF is mesothelial cells, we performed an angiogenesis tube assay. Briefly, HMRSV5 mesothelial cells were exposed for 3 h to PD or control solution and then recovered for 24 h. The conditioned mediums were used to culture endothelial cells in an angiogenesis assay.

**Figure 10:**
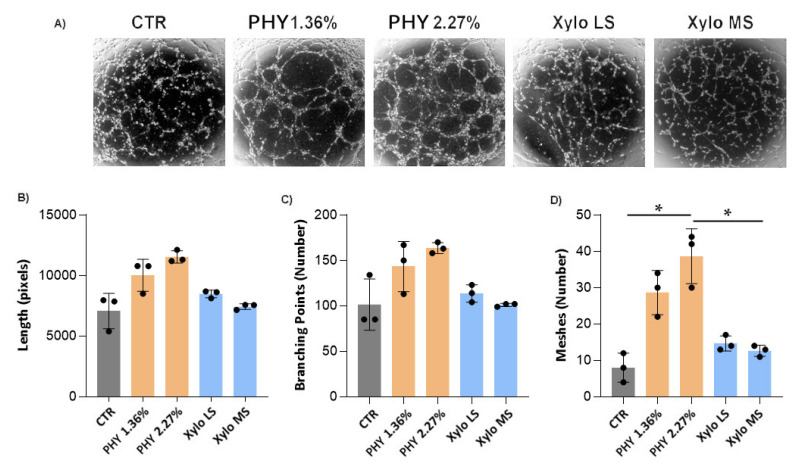
Angiogenesis assay. (**A**) The figure shows representative images of HUVEC endothelial cells, seeded on growth factor reduced matrigel, and cultivated in the presence of HMRSV5 mesothelial cell-conditioned medium. Morphometric characterization of the patterns generated by HUVEC cells on Matrigel is reported in the relative graphs. Data represent the dimensional [length (**B**)] and topological [branching points (**C**) and meshes (**D**)] parameters. Values are given as mean ± SD (*n* = 3 biological replicates). (**D**) CTR vs. PHY 2.27% *p* = 0.0023, PHY 2.27% vs. Xylo MS *p* = 0.0046 * *p* < 0.05.

HUVEC tube formation assay showed that the conditioned medium of mesothelial cells exposed to Physioneal induced a strong angiogenic response in endothelial cells as demonstrated by increase of the dimensional (length) and topological (branching and meshes) parameters. The angiogenic response induced by XyloCore was similar to control medium (Figure 10).

## 4. Discussion

The main aim of dialysis is to remove water and uremic solutes, and successful elimination of them is the key determinant of outcomes of patients treated with PD [50]. It is well-known that exposure to bio-incompatible PD solutions damages the peritoneal structure and over time this leads to loss of ultrafiltration capability, a condition associated with the development of fibrosis, inflammation, and angiogenesis [51], which causes technique failure [52]. It is therefore of critical importance to preserve a healthy peritoneum. Injury to the peritoneal membrane is due to recurrent episodes of peritonitis but mainly to the un-physiological composition of PD solutions [53]. Glucose-based solutions continue to be the most used in PD, although new, more biocompatible, less acidic solutions with lower GDP content have been developed over the years [54]. Although cell culture and animal studies have shown that the newer PD solutions achieve better biocompatibility than acidic glucose-based ones, this does not translate into clinical superiority [55,56,57,58,59]. Again, when one looks at the effect on biomarkers of angiogenesis and inflammation, VEGF and IL- 6 levels, there is no evidence of benefit [27,60,61].

Development of a more biocompatible, efficient, dialysate for use in the clinical practice is of utmost importance for the future of PD [62]. Novel approaches aiming to improve the biocompatibility of PD solution under initial clinical development are represented by the addition of cytoprotective agents, such as alanyl-glutamine or the use of osmo-metabolic agents, in the PD fluid.

The addition of the dipeptide alanyl-glutamine (Ala-Gln) to the PD dialysate has been shown in experimental models to protect from peritoneal membrane deterioration [63], to restore cytoprotective cell response [64], and to reduce PD-associated vasculopathy enabling protective processes [65]. Early clinical trials in man confirmed that the addition of Ala-Gln to glucose-based PD solutions restored the stress response and improved cellular host-defences in PD cells [66]. More recently, a double-blinded, randomized cross-over study examined the impact of Ala-Gln-containing PD fluid on biomarkers of peritoneal health [67]. The study included 50 PD patients, treated for eight weeks with Ala-Gln (8 mM) or placebo added to neutral pH, low GDP solutions. Results indicate that, differently from non-supplemented PD solutions, supplementation with ala-Gln could improve biomarkers of peritoneal membrane integrity, immune competence, and systemic inflammation [67].

Another novel strategy to improve the biocompatibility of PD solution is the osmo-metabolic approach, which is based on the use of compounds (osmo-metabolites) exhibiting both osmotically and metabolically favorable properties, like L-carnitine and xylitol [31]. The formulation of the novel PD solution (XyloCore) includes a low glucose amount (27.7 mmol/L), that did not seem to have the deleterious effects the higher concentration did [35], in order to take advantage of its UF ability. In a recent study [38], we compared the effects on mesothelial cells exposed only at the apical side (thus mimicking the condition of a PD dwell) of several different PD solutions including standard glucose-based, neutral ph low GDP (Physioneal, Bicavera), icodextrin, and amino acids [38]. Findings of the study shows better performance in terms of higher cell viability, better preservation of the integrity of the mesothelial layer, and reduced release of pro-inflammatory cytokines by the novel PD solution [38].

Here, we further investigated the biocompatibility of the new XyloCore solutions and their effect on fibrosis, inflammation, and angiogenesis, the main mechanisms that drive peritoneal failure.

Results showed that the short exposure to PD solution of cells grown on plastic or seeded on pored filters produced an almost comparable effect in terms of viability. We analyzed two mesothelial (HMRSV5 and MET5α) and two endothelial (HUVEC and HMVEC) cell lines. We observed that mesothelial and endothelial cells treated with XyloCore LS and MS maintained a viability comparable to control cells whereas glucose-based solutions, especially at higher osmotic strength, significantly reduced cell viability. To better mimic long-term/multiple cycle exposure to the PD solution of the peritoneal mesothelium of PD patients, we tested mesothelial cell viability after repeated exposures to pure PD solutions. We observed that, in comparison with Physioneal, the repetitive exposure to XyloCore significantly preserves mesothelial cell viability. Because of the pH of 7.0 and the presence of bicarbonate that partly replaces lactate as a buffer, our present results suggest that the effect on viability is mainly due to the glucose concentration rather than to the osmotic strength, pH and/or lactate levels. Actually, we observed significant differences in viability between the mesothelial cells treated with the two Physioneal solutions and no differences between those treated with the two XyloCore solutions. In line with this observation, we speculate that the mild loss of viability with XyloCore observed after multiple cycles of exposure is due to the small glucose content along with the potential protective effects of L-carnitine and xylitol.

Important evidence of the biocompatibility of a PD fluid is the preservation of epithelial-type structure and permeability in the mesothelial and endothelial cell layers. Optical microscopy shows that exposure to XyloCore preserves the mesothelial and endothelial phenotype. Upon exposure to Physioneal, especially at a higher osmotic strength, mesothelial and endothelial cells look elongated and lose cell-cell contacts. It has been proven that glucose reduces trans-epithelial resistance (TER) and increases mesothelial permeability [20]. Here, we confirm a reduction in TER after Physioneal treatment together with significantly increased permeability to albumin. To be noted that the use of both LS and MS XyloCore preserves TER and permeability of the mesothelial layer. Albumin lost with the peritoneal dialysate is an important clinical problem in PD patients. In fact, up to 8–9 g of proteins, primarily albumin, is lost daily, which may have an unfavorable impact on nutritional status and mortality [68].

A huge amount of literature shows that exposure of mesothelial cells to high glucose concentration and glucose-based PD solutions activates the EMT process [16]. Likewise, it has been proved that high glucose induces a similar process in endothelial cells called Endothelial-to-Mesenchymal Transition (EndoMT) [69]. A key fibrogenic factor involved in PD-associated peritoneal fibrosis is TGF-β [70]. High glucose and glucose-based solutions increase peritoneal production of this growth factor [71]. TGF-β activates several signaling (SMAD dependent and independent) pathways which lead to the modulation of gene transcription and activation of events like MMT/EndoMT, angiogenesis, and inflammation. In mesothelial and endothelial cells treated with XyloCore, the gene expression of TGF-β and SNAIL (its EMT-associated downstream transcription factor) is significantly lower than in cells treated with a glucose-based solution. During MMT/EndoMT, peritoneal mesothelial cells lose their epithelial phenotype and acquire new mesenchymal characteristics. The attenuation of MMT/EndoMT and associated fibrosis is important for maintaining PD functionality. Icodextrin and bicarbonate/low-GDP solutions have been introduced to prevent MMT/EndoMT induced by glucose-based PD solutions, though their efficacy is still uncertain [71,72,73]. Actually, our data show that glucose-based Physioneal induces MMT/EndoMT in mesothelial/endothelial cells as disclosed by the up-regulation of mesenchymal markers α-SMA and VIM and the down-regulation of epithelial/endothelial markers E-CAD/VE-CAD at both gene and protein level. Remarkably, the treatment of mesothelial and endothelial cells with XyloCore does not activate MMT/EndoMT. These findings indicate that XyloCore is a much more biocompatible solution which could contribute to reducing PD associated fibrosis of the peritoneal membrane.

Peritoneal injury causes activation of macrophages, neutrophils, endothelial and mesothelial cells, which are the main sources of pro-inflammatory cytokines including IL-6, IL-1β, IL-8, TNF-α, monocyte chemoattractant protein-1 (MCP-1), and macrophage inflammatory protein 2 [74,75]. The overproduction of these cytokines leads to an acute inflammatory response, neutrophil accumulation and mononuclear cell recruitment, resulting in exacerbation of the inflammation and sustaining peritoneal fibrosis and angiogenesis [76]. There is evidence that chronic inflammation of the peritoneal membrane deranges peritoneal solute transfers, which may have an unfavorable impact on PD clinical outcomes [77].

High glucose concentrations in PD solutions result in a proportionate increase in the intraperitoneal production of IL-6 [78] which has fibrogenetic activity via JAK/STAT3 signaling and TGF-b/Smad-3 pathways [79,80,81]. Here we confirm that glucose-based Physioneal PD solutions up-regulate *IL-6* expression in both endothelial and mesothelial cells and the increase is proportional to the osmotic strength (i.e., to the glucose concentration). On the other hand, the use of XyloCore (with lower glucose content) significantly reduces *IL-6* up-regulation at both osmotic strengths. Of note, while Physioneal treatment increases TGF-β expression and induces the fibrotic phenotype, XyloCore blunts TGF-β up-regulation and its down-stream effects. The fact that XyloCore has a lower impact on *IL-6* expression must also be taken into account. Since IL-6 is a key mediator in regulating early peritoneal response to infection, controlling both host defense and leukocyte trafficking during infections [82], the effect of XyloCore is potentially protective against the long-term effect of peritonitis, still a major problem in PD patients and a frequent cause of technique failure.

A second important element controlling peritoneal inflammation is the NOD-like receptor protein 3 (NLRP3)/interleukin (IL)-1β signaling pathway. It has been proposed that glucose-based PD fluids activate NLRP3/ASC complex, which in turn leads to caspase-1-mediated conversion of pro-IL-1β to IL-1β. Secreted IL-1β in turn enhances VEGF production and secretion, promoting microvascular permeability and angiogenesis [54,83,84,85]. Here we demonstrate that treatment with biocompatible glucose-based PD solutions increases *IL-1β* expression in mesothelial and endothelial cells and induces the production of active IL-1β in mesothelial cells. The present results show that XyloCore LS and MS do not influence IL-1β expression and production, giving an advantage in controlling PD associated inflammation and angiogenesis. Since TGF-β and IL-1β exert additive effects in the development of EMT in mesothelial cells [16], the low fibrogenic profile of XyloCore as compared to biocompatible glucose-based PD solution may also be due to the different effect on IL-1β production.

Mitochondria are central players in oxidative stress since mitochondrial ROS control EMT [49]. However, they activate an inflammatory response, which in a vicious cycle induce mitochondrial damage fueling a pathologic system [86]. Here, we confirmed that Physioneal at the higher glucose content increases mitochondrial oxidative stress whereas XyloCore does not suggesting a protective action on mitochondrial integrity.

In parallel with fibrosis, when exposed to long-term PD fluids, the peritoneum shows a progressive increase in capillary number (angiogenesis) [8,87], which contributes to ultrafiltration failure. It has been proposed that vascular endothelial growth factor (VEGF), a potent proangiogenic factor, has a crucial role in PD associated sub-mesothelial angiogenesis and functional decline [8,22,88]. VEGF is produced in response to multiple stimuli associated with PD [89] and is tightly interconnected with MMT since TGFβ too up-regulates VEGF [90]. VEGF is produced by both mesothelial and endothelial cells [91,92] and it has been shown that glucose-based solutions upregulate VEGF in PD patients [22]. In this study, we confirmed that Physioneal, especially at the higher osmotic strength (2.27% glucose), increases VEGF expression and secretion by mesothelial and endothelial cells. By contrast, XyloCore LS and MS induce only minimal VEGF up-regulation. We used conditioned medium from mesothelial cultures, exposed to glucose-based or XyloCore solutions, to test the angiogenic response of endothelial cells. The corresponding conditioned medium showed quite different angiogenic activities in HUVEC endothelial cells, very robust with those from biocompatible PD solution cultures, and none at all in those from XyloCore-exposed cultures.

Given the in vitro nature of our studies, to strengthen our observations, in vivo studies are required. However, preliminary results of the first clinical trial with XyloCore have shown that it was not only well tolerated with no significant adverse events, but also that dialysis efficiency, as well as fluid status, diuresis, and peritoneal ultrafiltration, was comparable to traditional glucose-based PD solutions [93].

## Figures and Tables

**Table 1:** Composition of peritoneal dialysis solutions.

	XyloCore		Physioneal 40	
*Osmotic Strenght*	Low Strength	Medium Strength	1.36%	2.27%
Xylitol mmol/L	46 (0.7% *w*/*v*)	98.6 (1.5% *w*/*v*)	0	0
Glucose mmol/L	27.7 (0.5%)	27.7 (0.5%)	75.5 (1.36%)	126 (2.27%)
L-Carnitine mmol/L	1.24	1.24	0	0
Sodium mmol/L	132	132	132	132
Calcium mmol/L	1.3	1.3	1.25	1.25
Magnesium mmol/L	0.5	0.5	0.25	0.25
Chloride mmol/L	101	101	95	95
Lactate mmol/L	35	35	15	15
Hydrogen Bicarbonate (mmol/L)	0	0	25	25
pH	5.5 +/− 0.5	5.5 +/− 0.5	7.0 +/− 0.5	7.0 +/− 0.5
Osmolarity mosmol/L	346.5	399.1	344	395

**Table 2:** Primer sequences used for reverse Real-Time PCR.

Gene		Primer Sequence (5′-3′)
*ACTB*	Forward	GGCGACGAGGCCCAGA
	Reverse	CGATTTCCCGCTCGGC
*a-SMA*	Forward	TACTACTGCTGAGCGTGAGA
	Reverse	CATCAGGCAACTCGTAACTC
*E-CAD*	Forward	TTCTGCTGCTCTTGCTGTTT
	Reverse	TGGCTCAAGTCAAAGTCCTG
*VE-CAD*	Forward	CAGCCCAAAGTGTGTGAGAA
	Reverse	TGTGATGTTGGCCGTGTTAT
*VIM*	Forward	AAAACACCCTGCAATCTTTCAGA
	Reverse	CACTTTGCGTTCAAGGTCAAGAC
*TGF-b*	Forward	CGTGGAGCTGTACCAGAAAT
	Reverse	GATAACCACTCTGGCGAGTC
*SNAI1*	Forward	AGTTTACCTTCCAGCAGCCCTAC
	Reverse	AGCCTTTCCCACTGTCCTCATC

## Supplementary Materials

The supplemental images are available online at https://www.mdpi.com/article/10.3390/nu13072282/s1, Figure S1: Cell viability, Figure S2: Mesothelial TER and permeability, Figure S3: *SNAI1* and *TGF-β* expression in mesothelial and endothelial cells, Figure S4: Expression of epithelial and mesenchymal markers in mesothelial cells, Figure S5: Expression of endothelial and mesenchymal markers in endothelial cells, Figure S6: Regulation of mitochondrial ROS in mesothelial cells, Figure S7: Uncropped Figure 6D, Figure S8: Uncropped Figure 8E, Figure S9: Uncropped Supplemental Figure S4D.
