# Supplementary material for: Biological Effects of XyloCore, a Glucose Sparing PD Solution, on Mesothelial Cells: Focus on Mesothelial-Mesenchymal Transition, Inflammation and Angiogenesis
Source: Nutrients. 2021 Jun 30;13(7):2282. doi: 10.3390/nu13072282 (PMC8308380; doi:10.3390/nu13072282)
Supplement: Supplementary file 1 [file nutrients-13-02282-s001.zip › nutrients-1258442-supplementary.pdf]

SUPPLEMENTARY FIGURES

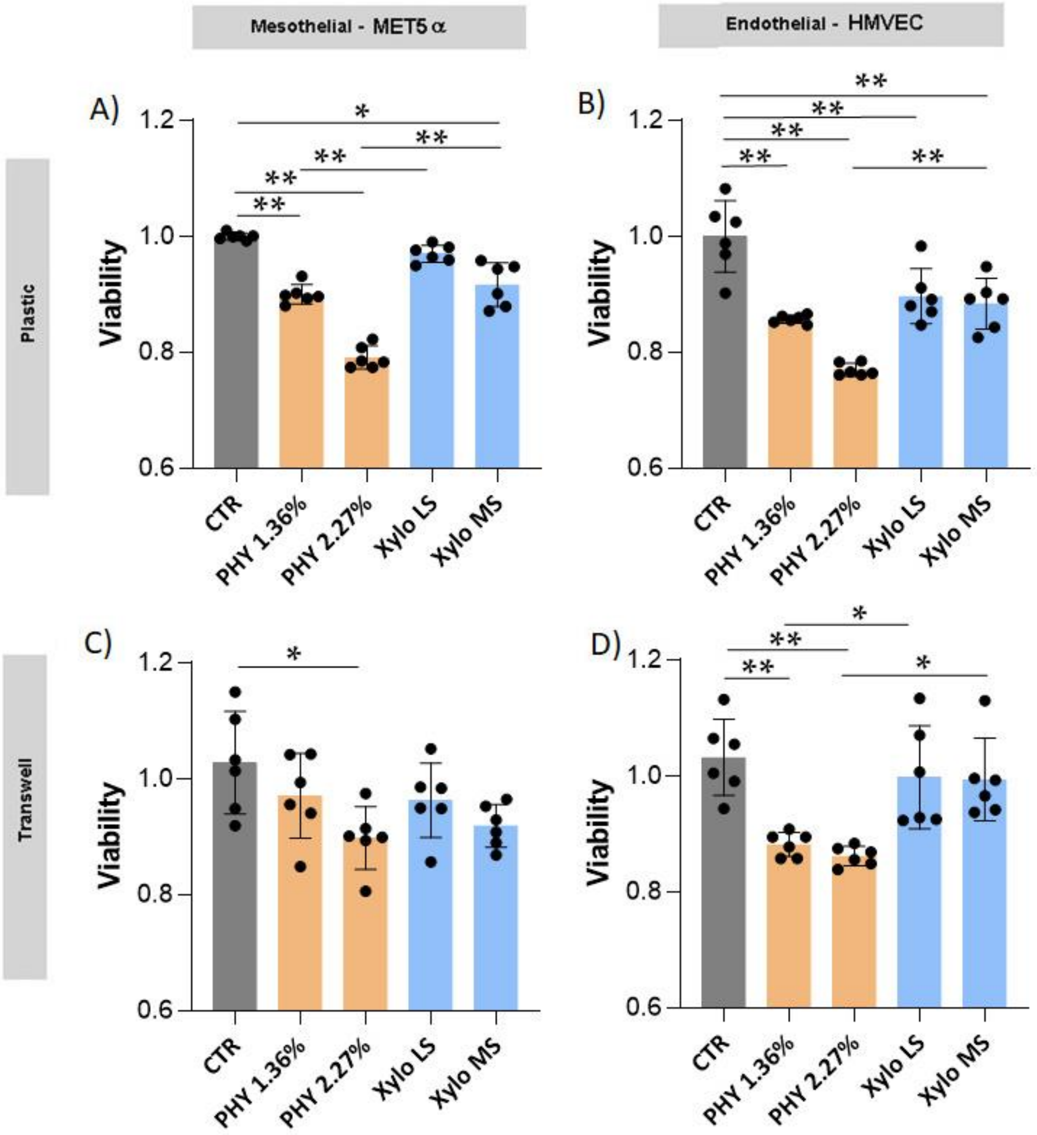

FIGURE S1: Cell viability. Cell viability was assessed with the MTS assay in MET5α mesothelial (A and C) and HMVEC endothelial (B and D) cells cultured in plastic (A and B) or transwell (C and D) with or without different PD solution. Mean ± standard deviation (error bars) (n=6 biological replicates). (A) CTR vs. FIX 1.36% p<0,0001, CTR vs. FIX 2.27% p<0,0001, CTR vs. Xylo MS p=0,012, FIX 1.36% vs. Xylo LS p<0,0001, FIX 2.27% vs. Xylo MS p<0,0001; (B) CTR vs. FIX 1.36% p<0,0001, CTR vs. FIX 2.27% p<0,0001, CTR vs. Xylo LS p=0,0018, CTR vs. Xylo MS p=0,0002, FIX 2.27% vs. Xylo MS p=0,0003; (C) CTR vs. FIX 2.27% p=0.0126; (D) CTR vs. FIX 1.36% p=0,0013, CTR vs. FIX 2.27% p=0,0003, FIX 1.36% vs. Xylo LS p=0,0157, FIX 2.27% vs. Xylo MS p=0,0049*P<0.05, **P<0.001 .

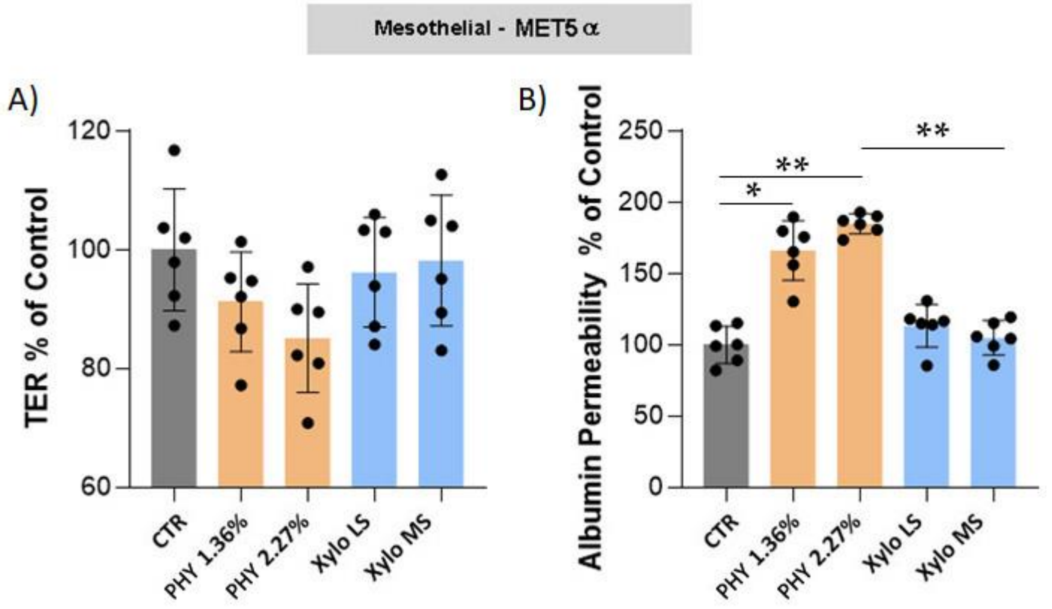

FIGURE S2: Mesothelial TER and permeability**.** (A) TER and (B) albumin permeability were measured in MET5α mesothelial cells grown with or without different PD solution. (B) CTR vs. FIX 1.36% p=0,0075, CTR vs. FIX 2.27% p<0,0001, FIX 2.27% vs. Xylo MS p<0,0001. *P<0.05, **P<0.001 (n=6 biological replicates)

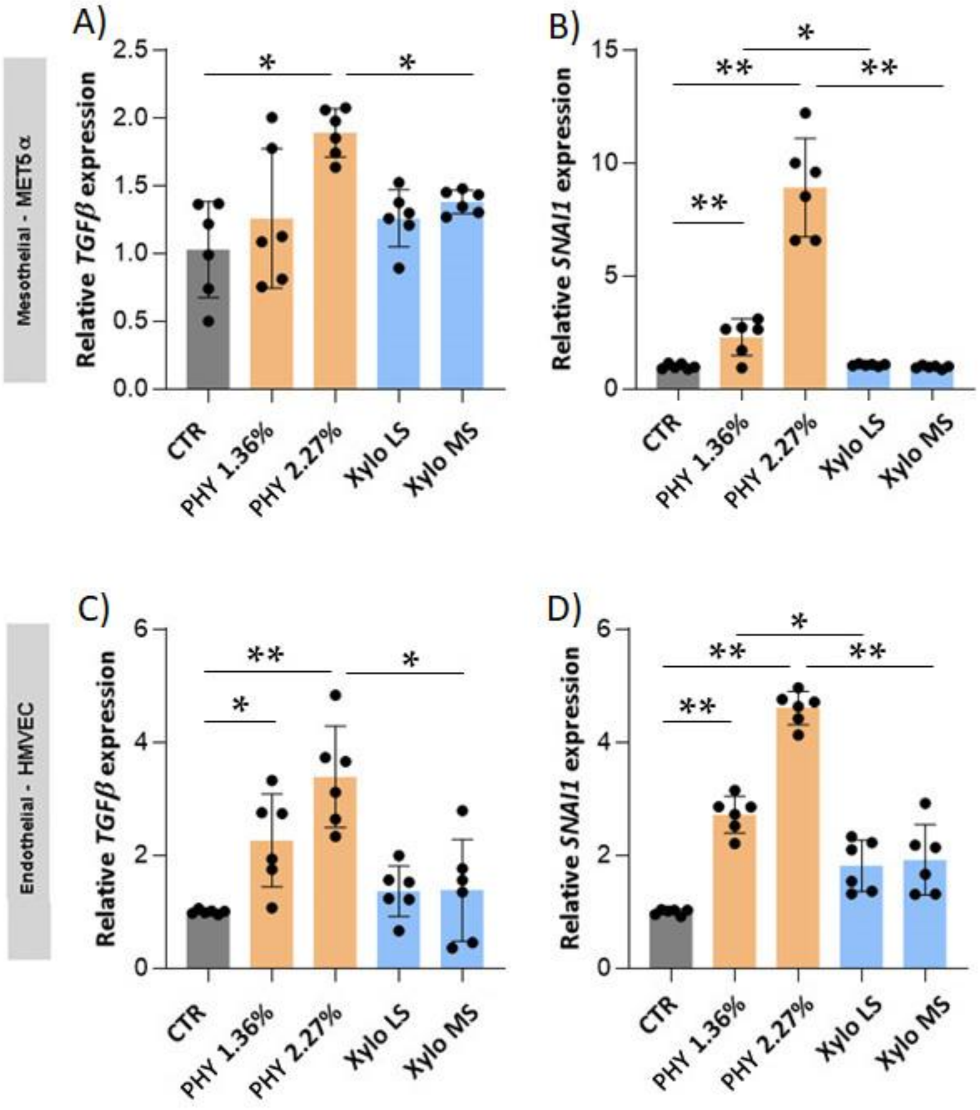

FIGURE S3: *SNAI1* and *TGF–β* expression in mesothelial and endothelial cells. *TGFβ* (A and C) and *SNAI1* (B and D) gene expression was quantified by real-time PCR. The analysis was performed on MET5α mesothelial cells (A and B) and HMVEC endothelial cells (C and D) treated for 3 hours in PD or control solution and then recovered with complete medium for 24 hours. The results were normalized using *ACTIN* as an internal control; they represent the mean ±S.D. (error bars) (n=6 biological replicates). (A) CTR vs. FIX 2.27% p=0,0030, FIX 2.27% vs. Xylo MS p=0,0425; (B) CTR vs. FIX 1.36% p=0,0216, CTR vs. FIX 2.27% p<0,0001, FIX 1.36% vs. Xylo LS p=0,0269, FIX 2.27% vs. Xylo MS p<0,0001; (C) CTR vs. FIX 1.36% p=0,0283, CTR vs. FIX 2.27% p<0,0001, FIX 2.27% vs. Xylo MS p<0,0001; (D) CTR vs. FIX 1.36% p<0,0001, CTR vs. FIX 2.27% p<0,0001, FIX 1.36% vs. Xylo LS p=0,0033, FIX 2.27% vs. Xylo MS p<0,0001. *P<0.05, **P<0.001 .

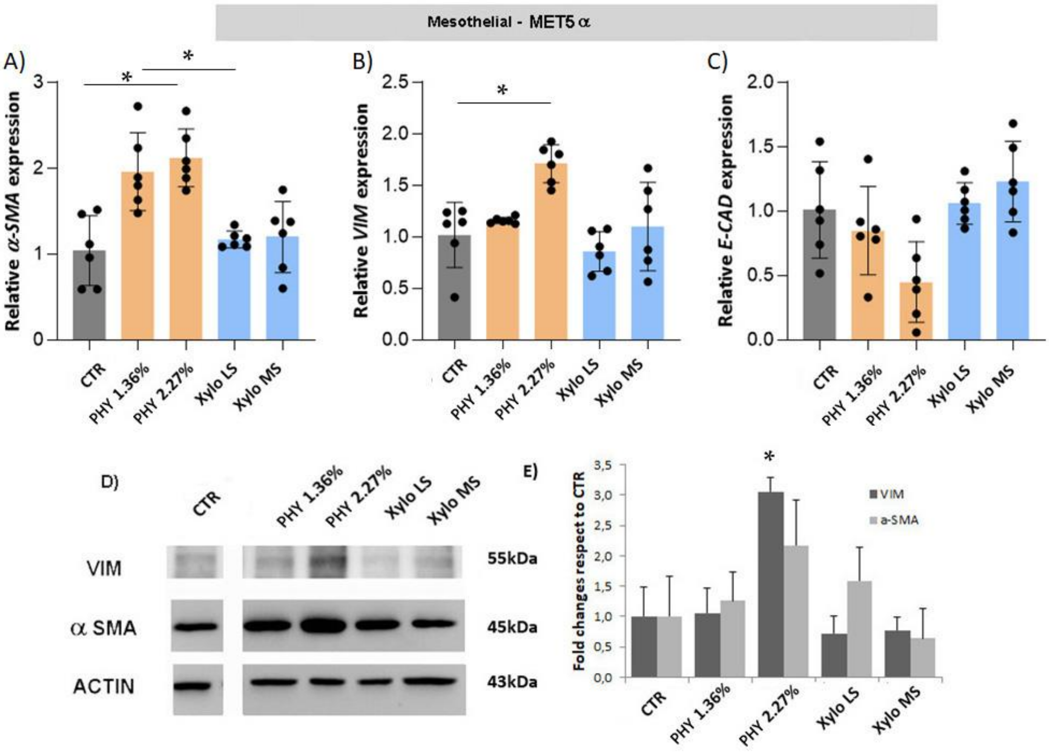

FIGURE S4: Expression of epithelial and mesenchymal markers in mesothelial cells. *α-SMA* (A), *VIM* (B) and *E-CAD* (C) gene expression was quantified by real-time PCR. The analysis was performed in MET5α mesothelial cells treated for 3 hours in PD or control solution and then recovered with complete medium for 24 hours. The results were normalized using *ACTIN* as an internal control. and represent the mean±S.D. (error bars) (n=6 biological replicates). (A) CTR vs. FIX 2.27% p=0,0022, FIX 1.36% vs. Xylo LS p=0,0056; (B) CTR vs. FIX 2.27% p=0,0018*P<0.05, **P<0.001. (D) Protein expression of α-SMA and VIM was evaluated by Western blot analysis. ACTIN was included as loading control. (E) WB quantification expressed as fold changes of bands intensity, normalized to ACTIN, respect to CTR (n = 3 biological replicates). Graphs represent mean ± standard deviation (error bars). *P<0.05.

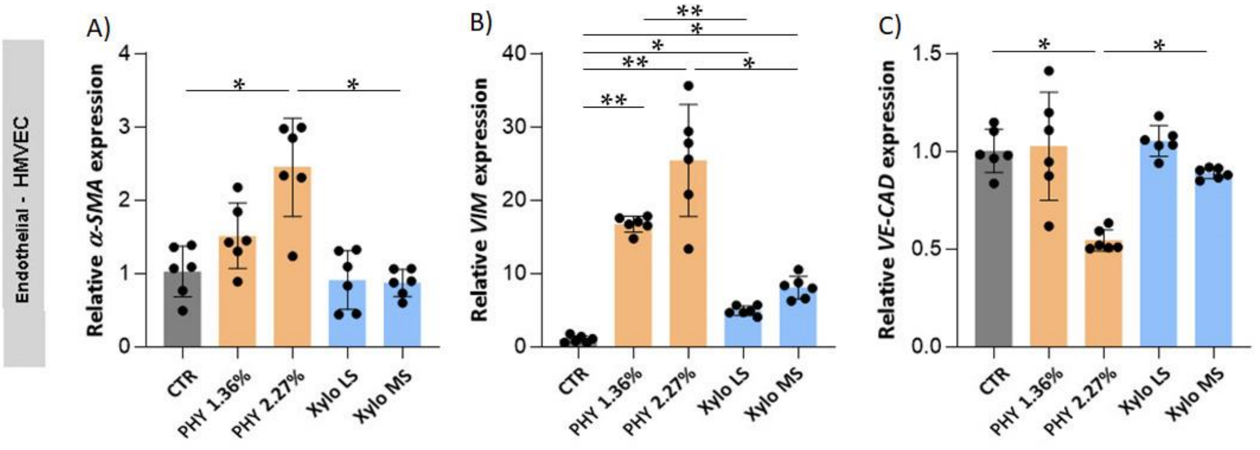

FIGURE S5: Expression of endothelial and mesenchymal markers in endothelial cells. *α-SMA* (A), *VIM* (B) and *VE-CAD* (C) gene expression was quantified by real-time PCR. The analysis was performed in HMVEC endothelial cells treated for 3 hours in PD or control solution and then recovered with complete medium for 24 hours. The results were normalized using *ACTIN* as an internal control and represent the mean±S.D. (error bars) (n=6 biological replicates). (A) CTR vs. FIX 2.27% p=0,0017, FIX 2.27% vs. Xylo MS p=0,0022; (B) CTR vs. FIX 1.36% p<0,0001, CTR vs. FIX 2.27% p<0,0001, CTR vs. Xylo LS p=0,0429,

CTR vs. Xylo MS p=0,0119, FIX 1.36% vs. Xylo LSp<0,0001, FIX 2.27% vs. Xylo MS p<0,0001; (C) CTR vs. FIX 2.27% p=0,0019, FIX 2.27% vs. Xylo MS p=0,0016 *P<0.05, **P<0.001.

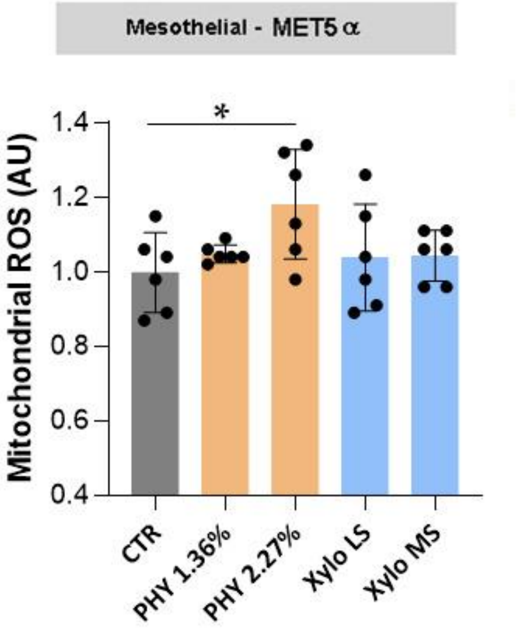

FIGURE S6: Regulation of mitochondrial ROS in mesothelial cells. Mitochondrial ROS was measured by MitoSOSred in MET5a cells treated for 3 hours in PD or control solution. Results are expressed as arbitrary units (AU) respect to CTR. Mean ± standard deviation (error bars) (n=6 biological replicates). CTR vs. PHY 2.27% p=0.0429.

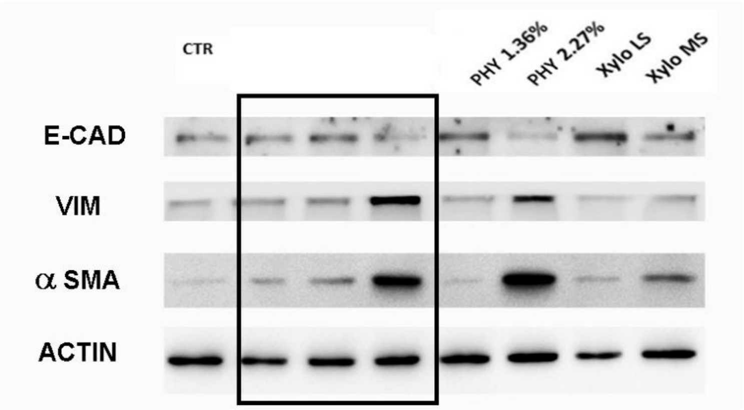

FIGURE S7: Uncropped Figure 6D

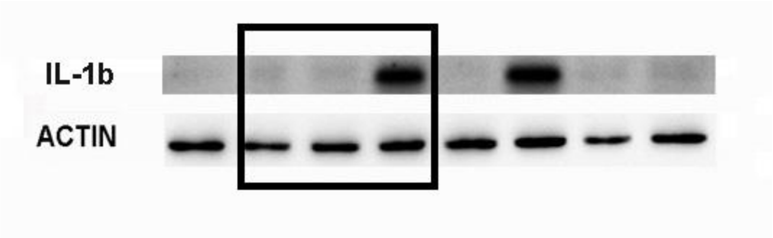

FIGURE S8: Uncropped Figure 8E

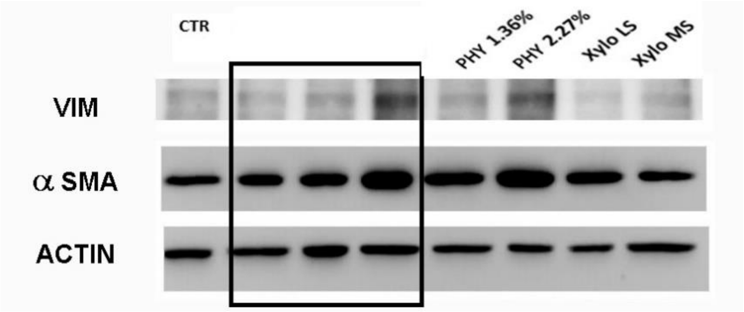

FIGURE S9: Uncropped Supplemental Figure 4D
